# *CHL1* hypermethylation as a potential biomarker of poor prognosis in breast cancer

**DOI:** 10.18632/oncotarget.15004

**Published:** 2017-02-02

**Authors:** Esperanza Martín-Sánchez, Saioa Mendaza, Ane Ulazia-Garmendia, Iñaki Monreal-Santesteban, Idoia Blanco-Luquin, Alicia Córdoba, Francisco Vicente-García, Noemí Pérez-Janices, David Escors, Diego Megías, Paula López-Serra, Manel Esteller, José Juan Illarramendi, David Guerrero-Setas

**Affiliations:** ^1^ Cancer Epigenetics Group, Navarrabiomed. Departmento de Salud-UPNA. IdiSNA, Pamplona, Spain; ^2^ Immunomodulation Group, Navarrabiomed. Departmento de Salud-UPNA. IdiSNA, Pamplona, Spain; ^3^ Department of Pathology, Complejo Hospitalario de Navarra, Servicio Navarro de Salud-Osasunbidea, Pamplona, Spain; ^4^ Department of Surgery, Complejo Hospitalario de Navarra, Servicio Navarro de Salud-Osasunbidea, Pamplona, Spain; ^5^ Confocal Microscopy Unit, Spanish National Cancer Research Centre, Madrid, Spain; ^6^ Cancer Epigenetics Group, Cancer Epigenetics and Biology Program, Bellvitge Biomedical Research Institute - IDIBELL, Barcelona, Spain; ^7^ Department of Physiological Sciences II, School of Medicine, University of Barcelona, Barcelona, Spain; ^8^ Institució Catalana de Recerca i Estudis Avançats (ICREA), Barcelona, Spain; ^9^ Department of Oncology, Complejo Hospitalario de Navarra, Servicio Navarro de Salud-Osasunbidea, Pamplona, Spain

**Keywords:** CHL1, DNA methylation, breast cancer, prognostic biomarker

## Abstract

The *CHL1* gene encodes a cell-adhesion molecule proposed as being a putative tumour-suppressor gene in breast cancer (BC). However, neither the underlying molecular mechanisms nor the clinical value of *CHL1* downregulation in BC has been explored. The methylation status of three CpG sites in the *CHL1* promoter was analysed by pyrosequencing in neoplastic biopsies from 142 patients with invasive BC and compared with that of non-neoplastic tissues. We found higher *CHL1* methylation levels in breast tumours than in non-neoplastic tissues, either from mammoplasties or adjacent-to-tumour, which correlated with lower levels of protein expression in tumours measured by immunohistochemistry. A panel of five BC cell lines was treated with two epigenetic drugs, and restoration of *CHL1* expression was observed, indicating *in vitro* dynamic epigenetic regulation. *CHL1* was silenced by shRNA in immortalized but non-neoplastic mammary cells, and enhanced cell proliferation and migration, but not invasion, were found by real-time cell analysis. The prognostic value of *CHL1* hypermethylation was assessed by the log-rank test and fitted in a Cox regression model. Importantly, *CHL1* hypermethylation was very significantly associated with shorter progression-free survival in our BC patient series, independent of age and stage (*p* = 0.001). In conclusion, our results indicate that *CHL1* is downregulated by hypermethylation and that this epigenetic alteration is an independent prognostic factor in BC.

## INTRODUCTION

Breast cancer (BC) is the most common cancer among women and one of the leading causes of cancer-related deaths worldwide [1–3]. It is a clinically heterogeneous disease, with at least five subtypes, according to the St Gallen International Expert Consensus in 2013 [4]: luminal A-like, luminal B-like/HER2-negative, luminal B-like/HER2-positive, HER2-positive (non-luminal) and triple-negative. Although BC incidence remains high, an increase in overall survival (OS) has been attributed to advances in early detection programmes and therapeutic approaches directed against molecular biomarkers, such as hormone receptors and HER2, which are overexpressed and amplified in luminal and HER2 subtypes, respectively. From the therapeutic point of view, their cell-signalling transduction abilities have been successfully abolished by administration of tamoxifen and trastuzumab, respectively [5–7]. Nevertheless, BC prognosis is quite variable and approximately 20-30% of early-stage cases will eventually experience recurrence and develop distant metastasis. Currently, however, there is no acceptable method for monitoring patients who are likely to progress [8]. BC is thought to result from the presence of certain abnormal genetic and epigenetic changes in tumour suppressor genes, such as *TP53* or *BRCA1*, and proto-oncogenes, like members of the PI3K signalling pathway, among others [4, 9]. A thorough understanding of the mechanisms responsible for BC development and progression is still needed to identify prognostic biomarkers.

Gene expression-based approaches have added significant prognostic and predictive value to pathological staging, histological grade and standard molecular marker identification [10]. However, the high cost of expression profiling and the molecular instability of mRNA have limited its clinical use, so expression-based BC classification has not become a routine method [11]. In fact, the most recent consensus [4] agreed a BC classification based on the expression of various immunohistochemical markers (presence or absence of oestrogen, progesterone and HER2 receptors and Ki67). Thus, although distinguishing BC subtypes by immunohistochemical markers is considered the gold standard, there is an urgent need to identify new and well-defined prognostic biomarkers to stratify BC patients with good and poor prognosis [12].

Epigenetic alterations are common molecular abnormalities in cancer, including DNA methylation, alterations in microRNA profiling and post-translational modifications of histones [13]. Over the past decade, aberrant DNA methylation has been recognised as one of the most common molecular abnormalities in BC [14, 15]. Methylation of certain genes has been related to clinical and pathological characteristics of breast tumours, and is considered a biomarker of diagnosis [16], hormone receptor [17] and HER2 [18] status, response to tamoxifen [17] and chemotherapy [19], metastases during follow-up [14], and has demonstrated its value as a predictor of survival [17, 20].

The *CHL1* gene (Close Homolog of L1, also known as *L1CAM2*, Entrez Gene accession number 10752) encodes a member of the L1 family of neural cell adhesion molecules essential for the brain development and involved in signal transduction pathways. Some of these proteins, such as L1CAM, are expressed in a wide range of tissues in addition to the brain, and are known to play an important role in carcinogenesis and progression in a variety of human cancers by overexpression and association with poor prognosis [2, 21]. Interestingly, *L1CAM* upregulation promotes cell adhesion and migration and is associated with shorter progression-free survival (PFS) and overall survival (OS) in BC [22–24].

However, very few studies have focused on the role of *CHL1* in cancer [2, 21]. There is weak evidence that *CHL1* expression is downregulated at the mRNA level in BC tissues relative to non-cancerous breast tissues [21], but nothing is known about the causes of this silencing. The biological role of *CHL1* in BC has been reported in only a single study, in which, in addition to confirming *CHL1* downregulation at the mRNA and protein levels in BC tissues and cell lines, the authors found that overexpression of *CHL1* impaired cell proliferation and invasion, while *CHL1* depletion caused the opposite effect *in vitro*, and promoted tumour formation *in vivo* [2]. Nevertheless, the clinical value of *CHL1* silencing in human tissues as a potential biomarker of prognosis remains to be elucidated. The aim of this study was to determine the mechanisms and clinical implications of *CHL1* downregulation in BC.

## RESULTS

### CHL1 hypermethylation is present in BC

**Figure 1:**
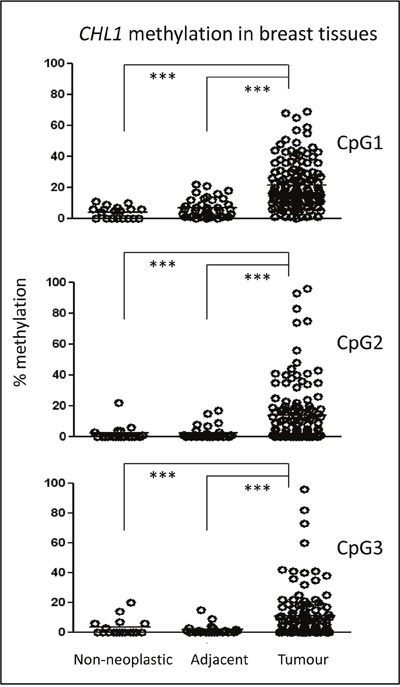
Epigenetic status of *CHL1* in BC patients The methylation of three CpG sites in the *CHL1* gene promoter was interrogated by pyrosequencing in a series of 142 BC cases, 45 adjacent-to-tumour tissues, and 19 non-neoplastic mammary tissues from reduction mammoplasties. The horizontal lines in each group represent the median of the series. (***, *p* < 0.001).

To determine the methylation status of the *CHL1* gene, three CpG sites in its promoter were pyrosequenced in a series of 142 breast tumours, 45 paired tumour and adjacent-to-tumour tissues, and 19 non-neoplastic breast tissues from reduction mammoplasties (Supplementary Figure 1). Since pyrosequencing provides a quantitative measure of methylation, the optimal cut-off value distinguishing statistically between the unmethylated and methylated status of each of the CpG sites was estimated by ROC curve analysis: 17.5% methylation for CpG1, 4.5% methylation for CpG2, and 9.5% for CpG3 (Table 1). We also considered that a case had hypermethylated *CHL1* when the three tested CpG sites simultaneously showed methylation percentages above their cut-off values. In contrast, non-neoplastic breast samples displayed very low percentages of methylation (< 11%) (Figure 1). Importantly, non-neoplastic adjacent-to-tumour tissues harboured significantly lower methylation levels in all CpG sites than tumour tissues, but slightly higher levels than those of non-neoplastic tissues (Figure 1). Interestingly, this epigenetic alteration was maintained across all BC subtypes (Supplementary Figure 2).

**Table 1:** Methylation status of *CHL1* in breast samples

	CpG1	CpG2	CpG3	All CpGs
Median % methylation in breast tumours (range)	18(0-69)	5(0-96)	5(0-96)	
Median % methylation in adjacent-to-tumour tissues (range)	6(0-22)	1(0-17)	1(0-15)	
Median % methylation in non-neoplastic breast samples (range)	5(0-11)	0(0-22)	0(0-20)	
Cut-off value (related to PFS)	17.5	4.5	9.5	Above cut-off in all CpGs

PFS: progression-free survival.

These results indicate, for the first time, that a subset of breast tumours has higher levels of *CHL1* gene methylation than do adjacent-to-tumour tissues and non-neoplastic samples.

### CHL1 protein expression pattern in mammary tissues

**Figure 2:**
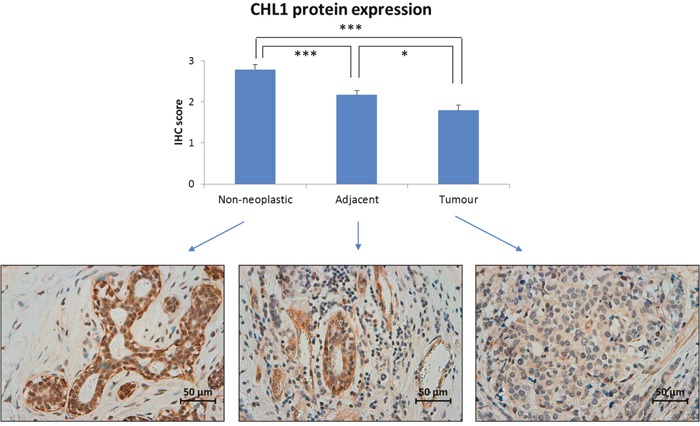
CHL1 protein expression in BC Immunohistochemistry was employed to measure CHL1 expression in 57 paired breast tumour and adjacent-to-tumour samples, along with 20 non-neoplastic tissues from reduction mammoplasties. It was scored as: 0, no expression; 1: weak expression; 2: intermediate expression; and 3: strong expression (***, *p* < 0.001; *, *p* < 0.05). Images were acquired at 400x magnification using a Leica DMD 108 digital microscope (Leica, Wetzlar, Germany).

Since DNA methylation is a well-known mechanism of gene expression regulation, the expression pattern of the CHL1 protein was measured by immunohistochemistry in 57 BC tissues, their adjacent-to-tumour counterparts and 20 non-neoplastic tissues from reduction mammoplasties. We found a significantly higher level of expression in both types of non-neoplastic cells relative to tumour cells, being slightly lower in adjacent-to-tumour than in non-neoplastic tissue (Figure 2 and Supplementary Figure 3A). Although the predicted location of CHL1 protein is the cell membrane, the pattern of expression was cytoplasmic without nuclear or membrane expression (Supplementary Figure 3B), even when using two different antibodies (data not shown). Furthermore, the same cytoplasmic pattern with a lack of membrane staining was observed by immunofluorescence in CHL1-expressing immortalized but non-neoplastic mammary cells (Supplementary Figure 3C).

These results are consistent with the epigenetic pattern we observed: breast tumours, with higher methylation levels, displayed a lower level of protein expression than did non-neoplastic tissues.

### CHL1 expression can be modulated by epigenetic drugs in BC cell lines

**Figure 3:**
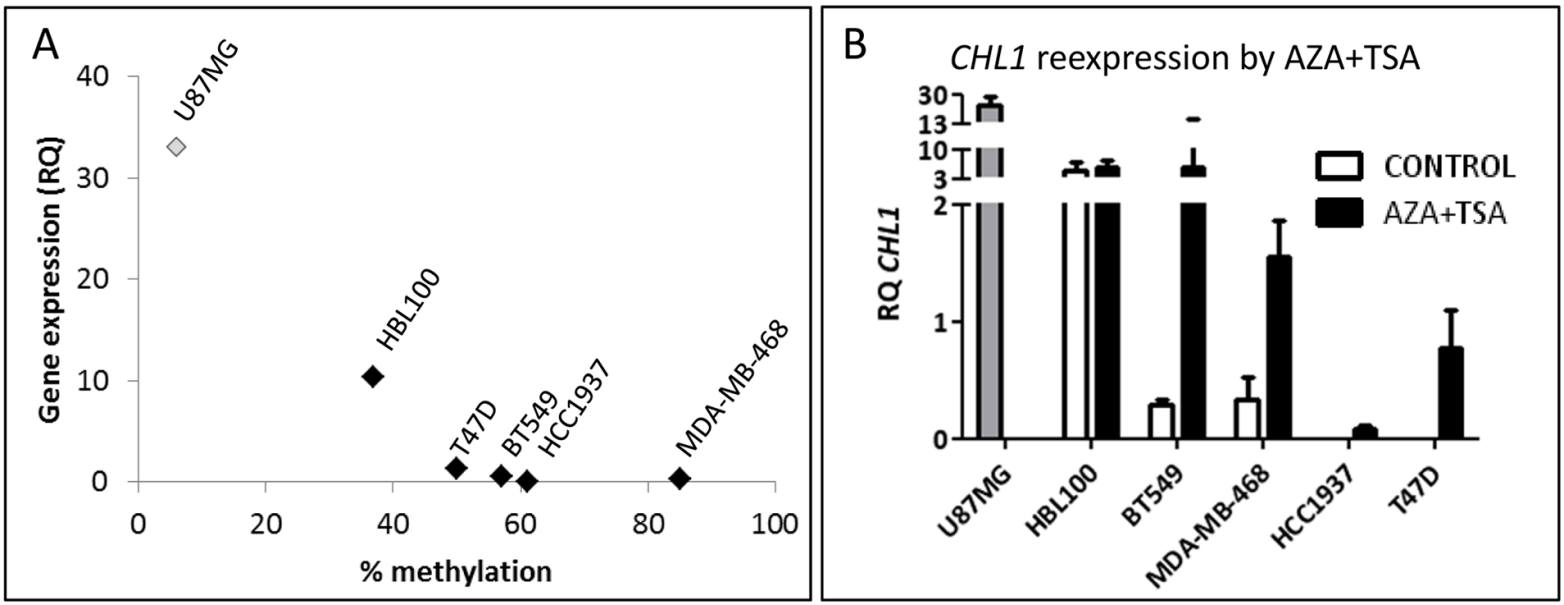
Molecular status of *CHL1* in BC cell lines **A**. Correlation between *CHL1* hypermethylation in the CpG1 site and expression in BC cell lines (Spearman's correlation coefficient = -0.9; *p* = 0.037), measured by pyrosequencing and qRT-PCR, respectively. U-87 MG cells were used as a positive control for *CHL1* expression, but were not included in the correlation analysis (RQ, relative quantification). **B**. Restoration of *CHL1* expression in BC cell lines by treatment with 4 μM 5-aza-dC for 72 h and 300 nM TSA for 24 h (AZA+TSA), measured by qRT-PCR.

The methylation status of the CpG1 site in the *CHL1* gene promoter that had been examined by pyrosequencing in BC samples was analysed in a panel of four BC cell lines and one immortalized but non-neoplastic mammary cell line. We found that the majority of BC cell lines had higher levels of methylation overall than non-neoplastic HBL-100 cells. Accordingly, mRNA levels of *CHL1* were lower in BC cell lines than in non-neoplastic HBL-100 cells, as assessed by qRT-PCR. In fact, we observed a strong and significant correlation between *CHL1* methylation and expression (Spearman's correlation coefficient = - 0.9; *p* = 0.037) (Figure 3A), suggesting that *CHL1* expression may also be regulated by methylation *in vitro* in BC.

In order to test whether *CHL1* expression can be modulated by epigenetic mechanisms, all cell lines were treated with two epigenetic drugs. We found by qRT-PCR that AZA+TSA treatment restored *CHL1* expression in all BC cell lines (Figure 3B), while single treatment was not as effective as the drug combination (data not shown).

These results indicate that the hypermethylation contributes to the regulation of *CHL1* expression in BC, and that it can be dynamically modulated by *in vitro* epigenetic treatments.

### CHL1 silencing promotes cell proliferation and migration of non-neoplastic mammary cells

**Figure 4:**
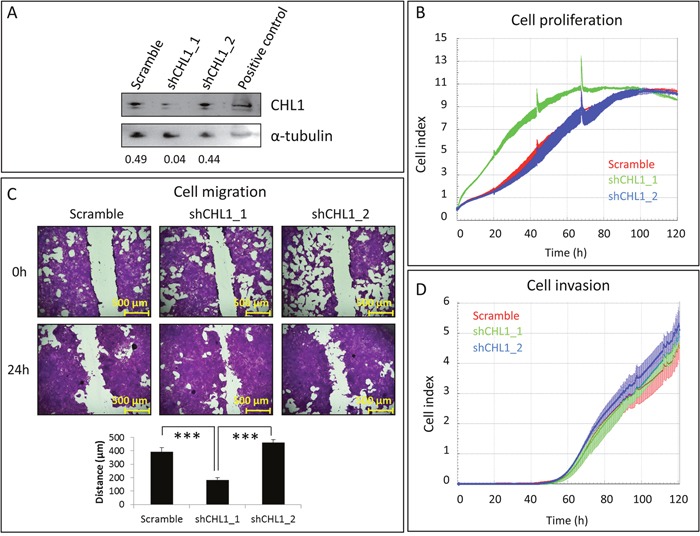
Effects of *CHL1* silencing on immortalized but non-neoplastic mammary cells **A**. HBL-100 cells were transduced with pHIV1-SIREN+scramble, pHIV1-SIREN+shCHL1_1, or pHIV1-SIREN+shCHL1_2 and selected with puromycin for 11 days. CHL1 silencing efficiency was checked by western blot, using α-tubulin as a loading control. Numbers indicate the amount of CHL1 relative to that of α-tubulin, as measured by densitometry. **B**. Cell proliferation was measured by RTCA for 5 additional days upon CHL1 silencing. **C**. Effects of CHL1 knockdown on cell migration were measured for 24 h. Images were acquired at 50x magnification with NIS-Elements, using an Olympus BX51 microscope (Olympus, Tokyo, Japan). **D**. Cell invasion was measured by RTCA for 3 additional days after CHL1 silencing.

To determine the effect of *CHL1* silencing in BC, we inhibited *CHL1* expression in the only mammary tissue-derived cell line expressing high levels of *CHL1*: the immortalized but non-neoplastic HBL-100 cell line. To this end, we inserted two shRNAs against *CHL1* and one scramble shRNA into the pHIV1-SIREN-PuroR plasmid, and lentiviruses were produced upon transfection in 293T cells. HBL-100 cells were then transduced and selected with puromycin. Western blot showed that the shCHL1_1 was more efficient at depleting CHL1 protein than shCHL1_2 (Figure 4A). Concomitantly, shCHL1_1, but not shCHL1_2, significantly enhanced HBL-100 cell proliferation (Figure 4B) and migration (Figure 4C), but not invasion (Figure 4D).

These observations indicate that *CHL1* silencing could be important for *in vitro* breast tumour cell growth.

### CHL1 hypermethylation predicts BC progression

**Figure 5:**
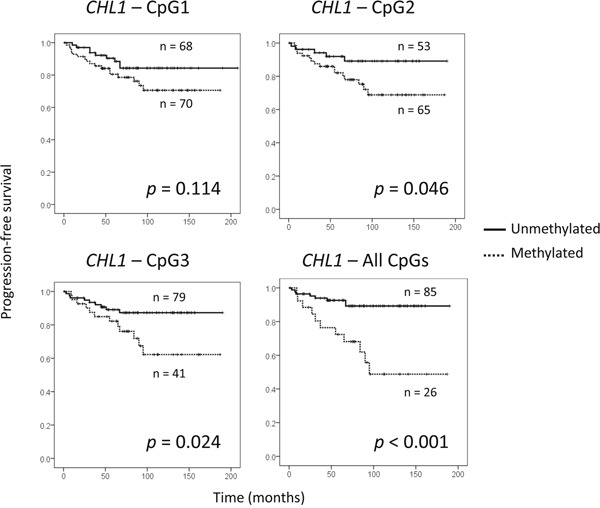
Prognostic value of CHL1 hypermethylation in a series of 142 BC patients (A) Association between shorter periods of progression-free survival and *CHL1* hypermethylation in each of the three CpG sites analysed, and in all of them simultaneously. Cut-off values for hypermethylation were calculated by ROC analysis: 17.5% for CpG1; 4.5% for CpG2; 9.5% for CpG3.

Finally, we aimed to examine the clinical value of *CHL1* hypermethylation in our series of 142 BC patients (Supplementary Table 1). Using the cut-off values of *CHL1* methylation mentioned above, we found that the methylation status of the *CHL1* gene was very significantly associated with shorter PFS (*p* < 0.001) (Figure 5), but not with OS (data not shown). We confirmed in our series that other well-known prognostic factors, such as lymph node involvement, histological grade and stage, substantially contributed to a shorter PFS (Supplementary Figure 4). Therefore, the independent impact of *CHL1* hypermethylation on progression, regardless of those important clinical variables, was tested in a Cox regression model. Importantly, we found that methylation in all the studied CpG sites in the *CHL1* promoter was still very significantly associated with poor PFS (*p* = 0.001), irrespective of age and stage (Table 2). The other clinical parameters significantly correlated with PFS (histological grade and lymph node involvement) were not included in the Cox regression model due to their association with the stage (*p* < 0.001). We also observed that the methylated status of all tested CpG sites of *CHL1* promoter displayed a hazard ratio of 5 (Table 2).

**Table 2:** *CHL1* hypermethylation as an independent prognostic factor

Variable	Hazard ratio(95% CI)	*p-value*
Age	1.012(0.970 – 1.055)	0.586
Stage	2.406(0.801 – 7.233)	0.118
*CHL1* hypermethylation – all CpGs	5.061(1.864 – 13.739)	0.001

Cox regression model shows the independent effect of each prognostic factor on progression-free survival (CI, confidence interval).

These results suggest that *CHL1* hypermethylation is of independent value as a predictor of shorter PFS in BC.

## DISCUSSION

The *CHL1* gene has been described as being downregulated in BC tissues with biological effects on cell proliferation in both *in vitro* and *in vivo* BC models [2, 21]. However, the mechanisms underlying *CHL1* silencing and its potential clinical role have not previously been explored. This gene is located on the short arm of chromosome 3 (band 3p26), a commonly deleted region in malignant peripheral nerve sheath tumours [25], nasopharyngeal carcinomas [26] and oral squamous cell carcinomas, in which the loss of this region is of prognostic value [27]. In BC, besides being deleted, this region has also been reported to harbour candidate tumour suppressor genes [21]. Deletions in one allele are usually accompanied by hypermethylation of the other [28]. In this study, we show for the first time that the *CHL1* gene, located in this region, is silenced in a subset of invasive BC cases due to promoter hypermethylation, but not in adjacent-to-tumour tissue and non-neoplastic tissue from mammoplasties. This epigenetic alteration has been found by pyrosequencing, a technique that yields quantitative measurements of methylation in contrast to methylation-specific PCR [29–33]. Consistent with the observed hypermethylation, we have found that tumour cells in BC tissues have a lower level of CHL1 expression compared with non-neoplastic adjacent-to-tumour cells. Importantly, this is the first report of the immunohistochemical pattern of CHL1 expression in invasive BC.

The biological role of *CHL1* silencing in BC was analysed in the only mammary tissue-derived cell line expressing high levels of *CHL1* mRNA available to us: an immortalized but non-neoplastic mammary cell line, HBL-100. All BC cell lines displayed very low *CHL1* expression, as previously described [2]. We also demonstrated that *CHL1* hypermethylation can be reversed by epigenetic treatment, since demethylating agents and histone deacetylase inhibitors modulated dynamics of *CHL1* expression *in vitro* and restored its silenced status in BC-derived cell lines. By reducing CHL1 protein levels with shRNA, a dramatic increase in non-neoplastic HBL-100 cell proliferation and migration was found (around 2-fold), but not in cell invasion, suggesting that *CHL1* might act as a tumour suppressor gene in the early stages of BC development. Interestingly, the expression of *L1CAM*, another neural cell adhesion molecule from the same family as *CHL1*, has been described to promote BC cell adhesion and migration *in vitro*, while cell invasion was also unaffected [24]. Our results are consistent with the only study to date to demonstrate the biological role of *CHL1* in BC [2], in which tumour cell proliferation and invasion were suppressed and stimulated by overexpression and depletion of *CHL1*, respectively, due to its interaction with the cytoskeleton [22]. The new *in vitro* finding observed in our study is that *CHL1* knockdown can also affect non-neoplastic cell proliferation, other than tumour cell spreading, as reported by He *et al* [2]. It has been proposed that during initial growth, *CHL1* is silenced in tumour cells to facilitate *in situ* tumour growth, acting as a tumour suppressor gene; *CHL1* is then re-expressed on the edge of the tumour mass and around tumour vessels to promote migration and local invasive growth, and acts as an oncogene to initiate the metastatic process [21].

The clinical role of *CHL1* hypermethylation in invasive BC has also been studied here for the first time. In this context, a cut-off value of *CHL1* hypermethylation has been established to stratify unmethylated and methylated cases, as seen with *MGMT* hypermethylation, which has been useful for predicting both PFS and OS in glioblastoma [34]. Importantly, we observed that *CHL1* hypermethylation was very significantly associated with shorter PFS in our large series of BC patients. Accordingly, an association between low *CHL1* mRNA levels and unfavourable histological grade was previously reported in a small series of breast tumours [2]. Most importantly, *CHL1* hypermethylation was an independent prognostic factor in our series that predicted shorter PFS, regardless of other crucial factors in BC prognosis, such as age or stage. Thus, testing *CHL1* hypermethylation by pyrosequencing, an easy-to-implement technique that returns an achievable and quantitative measurement [35], could have a significant clinical impact in BC patients.

In conclusion, our results show for the first time that *CHL1* promoter is hypermethylated in BC and that this epigenetic alteration, established with a quantitative cut-off value by pyrosequencing, is an independent prognostic biomarker in invasive BC.

## MATERIALS AND METHODS

### Patient samples

We analysed a series of 142 formalin-fixed, paraffin-embedded samples from BC patients alongside 20 non-neoplastic mammary samples from reduction mammoplasties. Paired adjacent-to-tumour tissue was available in 57 cases. All patients were diagnosed with primary invasive breast cancer between 1996 and 2006 in the Pathology Department of the Complejo Hospitalario de Navarra (Navarra Public Health System, Pamplona, Spain). Pathological and clinical characteristics are summarised in Supplementary Table 1. All tumours were surgically removed and staged according to their size, histological grade and lymph node involvement, and diagnosis was reclassified using the recommended criteria of the St Gallen International Expert Consensus in 2013 [4], considering a Ki-67 threshold of 14% [36], and upon microscopic evaluation by two independent observers with expertise in breast pathology. It was ensured that all cases harboured at least 70% tumour cells. None of the patients had received radiotherapy or chemotherapy before surgery. The study was approved by the Regional Clinical Research Ethics Committee and samples were obtained in accordance with the current Spanish legislation regarding written informed consent.

### Cell lines and treatments

A panel of four human BC cell lines was used in this study: T-47D (luminal-like) and BT-549 (triple-negative) were purchased from the American Type Cell Collection (ATCC, Rockville, MD, USA); HCC-1937 and MDA-MB-468 (all from the triple-negative subtype) were obtained from the Leibniz Institute DSMZ - German Collection of Microorganisms and Cell Cultures (Braunschweig, Germany). Additionally, one immortalized but non-tumorigenic human mammary epithelial cell line (HBL-100) was obtained from the ATCC (Rockville, MD, USA). Two cell lines derived from other tissues were used (all from ATCC, Rockville, MD, USA): human embryonic kidney 293T cells, which were used for transfection experiments; and U-87 MG, derived from human malignant glioma, which was used as a positive control for CHL1 expression. All these cell lines were grown in RPMI-1640 or DMEM, supplemented with 10% foetal bovine serum and 1% penicillin/streptomycin (all from Life Technologies, Carlsbad, CA, USA), at 37°C in a humidified atmosphere with 5% CO_2_.

All cell lines at low passage were treated with the demethylating agent 5-aza-2′-deoxycytidine (AZA) and the histone deacetylase inhibitor trichostatin A (TSA) (both from Sigma-Aldrich, St Louis, MO, USA). Briefly, cells were seeded at a density of 1×10^5^ cells/ml, allowed to attach overnight, and treated with 4 μM AZA for 72 h added freshly every 24 h, 300 nM TSA for 24 h, or the combination of the two drugs for the last 24 h, using PBS as a vehicle control.

### DNA extraction, bisulphite conversion and pyrosequencing

To determine the methylation status of the *CHL1* gene, DNA was extracted from formalin-fixed, paraffin-embedded breast tumours, non-neoplastic mammary tissues and BC cell lines using a QIAamp DNA FFPE Tissue kit (Qiagen, Hilden, Germany). Bisulphite conversion of DNA was performed to transform non-methyl cytosines into thymidines, while methyl cytosines remained intact. 500 ng of DNA were treated with freshly prepared bisulphite using an EZ DNA Methylation-Gold kit (Zymo Research, Irvine, CA, USA), following the manufacturer's instructions. Pyrosequencing was carried out to analyse the methylation of three CpG sites in the promoter of the *CHL1* gene (Supplementary Figure 1). For this purpose, first, PCR amplification was performed using Immolase DNA polymerase (BioLine, London, UK) in a final volume of 30 μl containing 2 μl of bisulphite modified DNA and the primers indicated in Supplementary Table 2. Amplification conditions were: initial DNA polymerase activation at 95°C for 10 min, followed by 50 cycles at 95°C for 30 s, 58–60°C for 30 s and 72°C for 30 s, and a final extension at 72°C for 7 min. The amplicons were resolved by electrophoresis using 2% (w/v) agarose gel in 1x Tris-borate-EDTA buffer, stained using SYBR Red Safe (Life Technologies, Carlsbad, CA, USA) and visualized in a standard transilluminator (ChemiDoc XRS, Bio-Rad Laboratories, Hercules, CA, USA). DNA methylation analysis was quantified as follows: 20 μl of PCR products were immobilized with Streptavidin Sepharose HP Beads (GE Healthcare Bio-Sciences, Pittsburgh, PA, USA) using a Vacuum Prep Workstation. This was followed by annealing (80°C for 2 min) the sequencing primers (Supplementary Table 2) and pyrosequencing in a PyroMark Q24 using PyroMark Gold Q24 reagents and PyroQ-CpGTM Software (v.1.0.11) (all from Qiagen, Hilden, Germany). Results were analysed using PyroMark Q24 software in CpG analysis mode.

### Immunohistochemistry

3-μm sections of 57 BC tumours and their matched adjacent-to-tumour counterparts, along with 20 non-neoplastic tissues from reduction mammoplasties, were placed on slides and then deparaffinized, hydrated and treated to block endogenous peroxidase activity. After incubating with the primary rabbit polyclonal CHL1 antibody (ab106269, Abcam, Cambridge, UK) at 1:800 dilution for 20 min (antigen retrieval at 90°C for 20 min, pH = 6.0), the antibody was developed using a Bond Polymer Refine Detection kit (Leica, Wetzlar, Germany) and visualized with diaminobenzidine. The pattern of expression was evaluated blind by two independent observers. The intensity of expression was ascribed to one of four categories: 0, no expression; 1, weak expression; 2, intermediate expression; 3, strong expression. Images were acquired with a Leica DMD 108 digital microscope (Leica, Wetzlar, Germany).

### Immunofluorescence

In order to explore the CHL1 expression pattern in cultured cells, the immortalized but non-neoplastic mammary HBL-100 cells were seeded on coverslips and allowed to attach overnight. Then, cells were fixed with 4% paraformaldehyde, blocked with 5% foetal bovine serum in PBS at room temperature for 1 h, and incubated with anti-CHL1 primary antibody (ab106269, Abcam, Cambridge, UK) at 4°C overnight at 1:800 dilution, and with AlexaFluor 488 secondary anti-rabbit (1:200) and AlexaFluor 594 Phalloidin (1:500) (both from Life Technologies, Carlsbad, CA, USA) at room temperature for 1 h. Finally, samples were mounted on slides with DAPI to counterstain nuclei. Confocal microscopy was performed with a Leica TCS SP5 laser scanning microscope (AOBS) (Leica, Wetzlar, Germany) using excitation wavelengths of 488 nm (for FITC) and 561 nm (for Texas Red).

### RNA extraction and quantitative reverse transcription PCR (qRT-PCR)

qRT-PCR was performed to measure the levels of *CHL1* expression in BC-derived cell lines and to check the restoration of gene expression by AZA+TSA treatment. To this end, first, total RNA was extracted and purified using an RNeasy Mini Kit (Qiagen, Hilden, Germany) following the manufacturer's instructions. 500 ng of total RNA were retrotranscribed using a PrimeScript™ RT Reagent Kit (TaKaRa, Otsu, Japan) at 37°C for 15 min and 85°C for 5 s. 1 μl of the resulting cDNA was placed in a 96-well plate with 0.5 μl TaqMan probes (*CHL1*: Hs00544069_m1 from Life Technologies, Carlsbad, CA, USA; and *GAPDH*: Hs.PT.39a.22214836, from IDT, Coralville, Iowa, USA) and 19 μl of mix were included in the Premix Ex Taq™ kit (TaKaRa, Otsu, Japan). PCR amplification was performed in triplicate using the Quant Studio 12K Flex (Life Technologies, Carlsbad, CA, USA) under thermal cycler conditions of 95°C for 30 s and 40 cycles at 95°C for 5 sec and 60°C for 34 s. The cycle threshold (Ct) values were calculated using Quant Studio software (Life Technologies, Carlsbad, CA, USA), and the relative quantification (RQ) was calculated by the ΔCt method (RQ = 2^−ΔCt^), using *GAPDH* as the endogenous control gene.

### CHL1 silencing in immortalized but non-neoplastic mammary cells

To study the functional role of *CHL1* in BC, HBL-100 cells were transduced using lentivirus containing short-hairpin RNAs (shRNAs) against *CHL1*. For their construction, two sequences targeting *CHL1* (shCHL1_1: 5’-GCAGCAATATTAGCGAGTATAT-3’ and shCHL1_2: 5’-GCGTCCATTGATACAAACCAAA-3’) and one scramble sequence (5’-GCAACAAGATGAAGAGCACCAA-3’) were inserted into the pHIV1-SIREN-PuroR plasmid [37] through digestion with *BamHI* and *EcoRI* restriction enzymes (Life Technologies, Carlsbad, CA, USA) and ligation with the T4 DNA ligase enzyme (New England Biolabs, Ipswich, MA, USA). Plasmids were purified using the Qiagen Plasmid Midi kit (Qiagen, Hilden, Germany) and sequenced to check the ligation. Lentiviruses containing the scramble, shCHL1_1 or shCHL1_2 were produced by the three-plasmid cotransfection method in 293T cells: p8.91, encoding HIV-1 structural proteins; pVSVg, encoding the vesicular stomatitis virus surface glycoprotein; and the constructed plasmids (scramble, shCHL1_1 or shCHL1_2). Media containing viruses were recovered and filtered every day for a week, ultracentrifuged at 25,000 rpm at 4°C for 2 h and stored at -80°C until used. Since the plasmid contains the puromycin resistance gene for mammalian cell selection, sensitivity to this antibiotic was first tested in HBL-100 cells, and an optimal concentration of 1 μg/ml was chosen from a wide range of possibilities. HBL-100 cells were then transduced with 5 μl of each lentivirus for 24 h (multiplicity of infection: 2.5 lentiviral particles/cell), and once the lentiviral particles had been removed, puromycin was added to the culture medium and cells were maintained for 2 weeks for selection.

### Western blot

*CHL1* silencing efficiency was checked by western blot. Upon puromycin selection, cells transduced with the scramble, shCHL1_1 or shCHL1_2 were harvested, lysed with 30 μl of RIPA buffer (Sigma-Aldrich, St Louis, MO, USA) containing a cocktail of protease inhibitors (Roche, Basel, Switzerland). After centrifugation at 8000 x *g* for 10 min at 4°C, proteins contained in the supernatants were quantified using the Protein DC kit (Bio-Rad, Hercules, CA, USA) in an Epoch plate reader (BioTek, Winooski, VT, USA) and following manufacturer's recommendations. For western blot, 80 μg of proteins were resolved by SDS-PAGE in a 10% gel and transferred to a nitrocellulose membrane (Bio-Rad, Hercules, CA, USA). The membrane was blocked with 5% non-fat milk and incubated with the anti-CHL1 antibody (ab106269, Abcam, Cambridge, UK) at a 1:500 dilution, overnight and at 4°C. It was then incubated with the secondary anti-rabbit antibody (Bio-Rad, Hercules, CA, USA) at 1:3000 for 1 h at room temperature. The signal was detected with the SuperSignal West Pico Chemiluminiscent Substrate kit (Thermo Scientific, Rockford, IL, USA) in a ChemiDoc (Bio-Rad, Hercules, CA, USA) using the ImageLab software. The α-tubulin antibody (T-6074 from Sigma-Aldrich, St Louis, MO, USA) and the secondary anti-mouse antibody were used at 1:10000 and 1:2000, respectively, for 30 min, as a loading control. Finally, the intensity of bands was quantitated by densitometric analysis using ImageJ software.

### Real-time cell analysis (RTCA) of cell proliferation and invasion

To evaluate the functional role of *CHL1* in cell proliferation, HBL-100 cells transduced with scramble and two shCHL1 were seeded (1×10^4^ cells/well) into 400 μl of medium in an E-plate L8 device (iCELLigence system, ACEA Biosciences, San Diego, CA, USA), after measuring the background in 100 μl of medium. The invasion assays were performed in CIM-16 plates with 8-μm-pore membranes (ACEA Biosciences, San Diego, CA, USA). Wells were coated with 30 μl of 5% Matrigel (BD Biosciences, San Jose, CA, USA) and allowed to gel at 37°C and 5% CO_2_ for 4 h. Then, the lower chamber wells were filled with 160 μl of medium containing 10% foetal bovine serum and the top chamber wells with 40 μl of serum-free medium. The two portions were assembled together and allowed to equilibrate for 1 h at 37°C and 5% CO_2_. Cells were incubated for 16 h in 0.05% foetal bovine serum media; for seeding, the cells were rinsed with PBS, trypsinized and resuspended in serum-free medium. A total of 4 × 10^4^ cells/well were seeded onto the top chamber of CIM-16 plates and placed into the xCELLigence system (ACEA Biosciences, San Diego, CA, USA) for data collection after background measurement.

Two replicates for each condition were analysed. Cell attachment, spreading, proliferation and invasion were monitored by RTCA for 3-5 days, on the basis of changes in cell-sensor impedance, as previously described [33, 38].

### Cell migration

To examine the role of CHL1 silencing on cell migration, HBL-100 cells transduced with the scramble, shCHL1_1 and shCHL1_2 were seeded into 6-well plates at a density of 2×10^5^ cells/well. When they had nearly reached confluency, cells were serum-starved for 8 h, then three scratches were made in the cell monolayer with a 10-μl pipette tip, and cells were washed twice with PBS 1X. Some cells were harvested here (time, 0 h), while others were maintained for 24 h in a culture medium containing 5% foetal bovine serum, as previously described [22]. Finally, harvested cells were fixed with paraformaldehyde and stained with crystal violet (Sigma-Aldrich, St Louis, MO, USA), and 10 pictures were taken with an Olympus BX51 microscope (Olympus, Tokyo, Japan). The length of the scratch in each picture was determined using NIS-Elements software from more than 10 measurements taken from each picture.

### Statistical analysis

Demographic, clinical and pathological data were summarised as frequencies (and percentages) and means or medians (and ranges) ± standard error of the mean, as appropriate. All statistical analyses were carried out using IBM SPSS Statistics v20. The optimal cut-off values identifying the methylated or unmethylated status of each CpG were estimated using ROC curve analysis. Across several cut-off points, Youden's index was chosen as the best cut-off value by considering maximum sensitivity and specificity. The optimal cut-off values predicting OS and PFS were estimated as previously described [34]. Methylation levels in tumour, adjacent-to-tumour and non-neoplastic tissues were compared using the Kruskal-Wallis test. Methylation in tumours and their adjacent counterparts was compared by a paired t-test. Differences in immunohistochemical expression were analysed with the Mann-Whitney and Wilcoxon tests. Correlation between methylation and expression in BC cell lines was assessed by Spearman's correlation coefficient: U-87 MG cells were excluded from this analysis. The effects of *CHL1* silencing on cell proliferation, migration and invasion were compared using two-tailed unpaired t-tests at different times (0, 24, 48, 72, 96, 120 h). Finally, Kaplan-Meier plots and log-rank tests were used to examine the association of *CHL1* hypermethylation with PFS and OS. A multivariate Cox regression model was fitted to test the independent contribution of each variable to patient outcome. Hazard ratios and 95% confidence intervals were used to estimate the effect of each variable on the outcome. Association between clinical variables was tested with the χ^2^ test.

## SUPPLEMENTARY FIGURES AND TABLES


