# Supplementary material for: CHL1 hypermethylation as a potential biomarker of poor prognosis in breast cancer
Source: Oncotarget. 2017 Feb 2;8(9):15789–801. doi: 10.18632/oncotarget.15004 (PMC5362523; doi:10.18632/oncotarget.15004)
Supplement: Supplementary file 1 [file oncotarget-08-15789-s001.pdf]

CHL1 hypermethylation as a potential biomarker of poor prognosis in breast cancer

SUPPLEMENTARY FIGURES AND TABLES

```
taaaagaaattattttagtagtataatttaaatttaggataaatttgttttatttgtatttataattt
gttaaggtagttggtaagaattgtgtttatatataaattgatagaatcgaggttttaaggttagcgatga
ttaaaatttaaaagtttatttatttattttaaaatatttattcgattgtttttgaggtagaattatga
gttgttttcgtttttgttgttttcggtggtggtatttaattttgaggatagtttatttaattttgagggt
agttttgaggggagttaaatattttttattgtttaatgagttagatgaaaataaaacgaggttgtaaggtt
aattttatagttttagtggagtaaaaattagaagttaataattttttattaataaagaggatttgtatt
taataggattttaaaagtggtatttgtaaaaattttggaaaatattattataatggaaaaaaaataaa
gtggtatataaattaaatgtagtgttaaaaattataatttttttatatgaattttggaattttaaaatat
ttttatgtgtttgtggatttataaggtattagaattggtttttgtattgaatatgagtatagaaggttt
ttttttttaatagtgaattaataaaggataagtaggaaaaacgttacgaaacgatgtggtaatattatag
attttgtattagggtaaaaattgtgagatagtagaagatttgtaaatttaagtatgtataatttgatagc
ggatggtcgtagtatatttttgtaaatttaatagtaagttttattacgttttttaaatgaaggaaagtaaga
agataatttttaatacgtatatggtattatatttttttaagacgtagtaatgggagaaaagtagattg
gcgatttgtgtgtgtaatatgaatcgagtgaaattatcggggaggggtgggggcgttttttta
aatgtcgttttgtagataaacgagtAGGGATTTTATTTTTTTTGATCGGAGATGTTGAAAAGTACGATT
AAAGTTTTTGGTTTGGTTTTTTGTTTTTTATAACGAGATAAAGTTTGAAGTTTGTAATTTTTTTTGTTTC
GCGGGAGCGTGTAAAGGGAGGGAAGATAAGCGGCGTGGGTGAGGGGTGGCGGCGTAGAATTTAGGAAGGG
AGTAGAGGCGAGAGTTAGTTTTCGGTAGGAGGGCGTAGATTTCGGTGTTAGTTAGAACGGTTTTAGTTTT
TTCGCGGTTAGAGAGTTAGCGGCGGGAGGGGACGGGCGGGATTTTCGCGGATAGTTTCGGGTGCGGATTA
GCGGGTAGCGGTCGGCGAGATTTTTTTTGGATTCGTTTAGAGTTTATCGGATTTTGCGCGTTTTCGTTTC
GGTTTTCGGTCGGTTCGGGGGAGAAGGCGTTCGAGGGGAGGCGTCGGATAGATCGCGTTTCGGAGGCGGC
GTAG
```

Forward primer CpG1	Forward primer CpG2 and CpG3
Sequencing primer CpG1	Sequencing primer CpG2 and CpG3
Reverse primer CpG1	Reverse primer CpG2 and CpG3

Supplementary Figure 1: The *CHL1* gene. Bisulphite-converted sequence of the *CHL1* promoter (lower case) and the first exon (upper case). The three CpG sites examined in this study are highlighted.

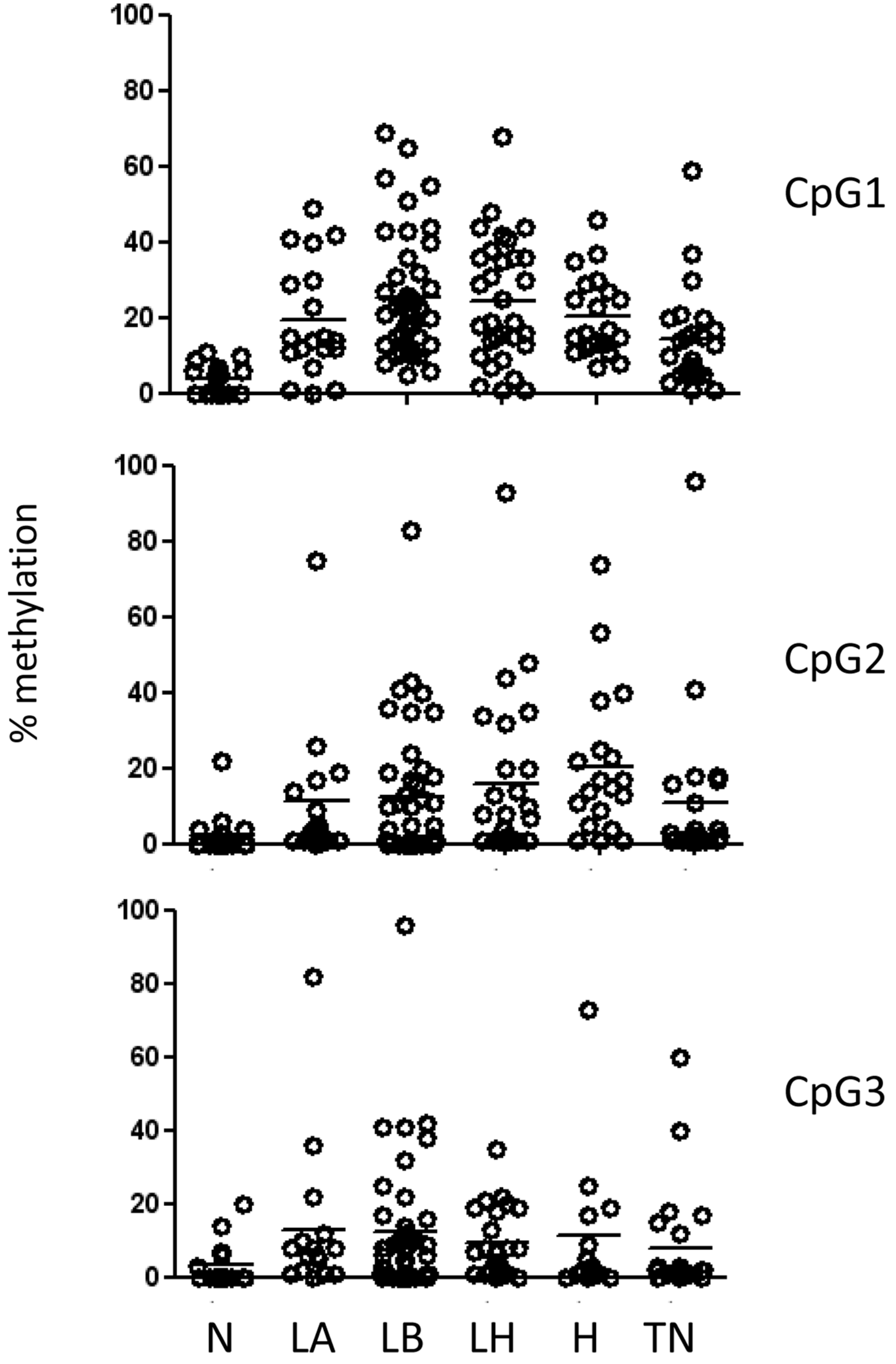

Supplementary Figure 2: Methylation status of *CHL1* promoter in BC subtypes. (A) Levels of methylation of three CpG sites were measured by pyrosequencing in our series of 142 BC cases, classified into five major subtypes: LA, luminal A (n=20); LB, luminal B/HER2-negative (n=44); LH, luminal B/HER2-positive (n=33); H, HER2-positive (n=21); TN, triple-negative (n=24); and N, non-neoplastic mammary tissues from reduction mammoplasties (n=19). The horizontal line indicates the median of each group.

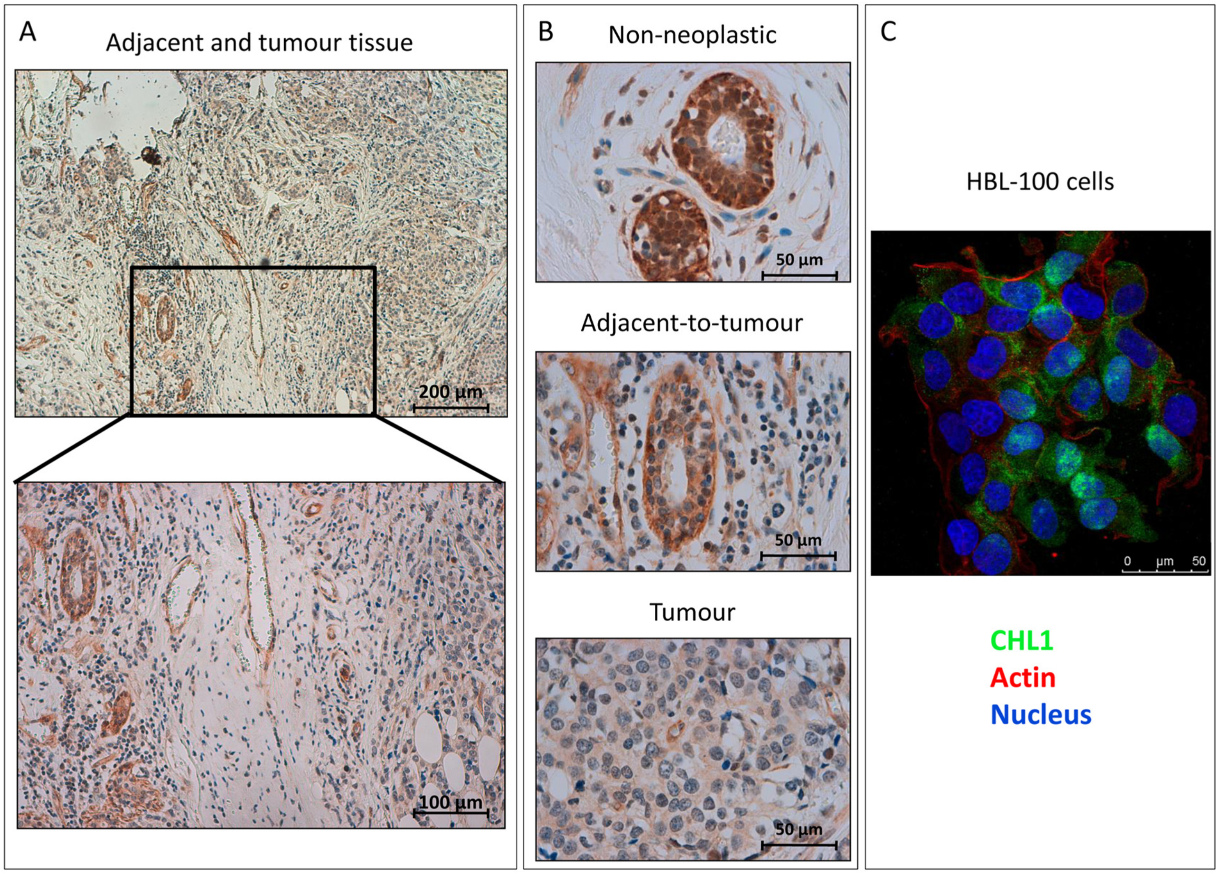

Supplementary Figure 3: CHL1 protein expression pattern in BC. Representative pictures of mammary tissues immunostained with CHL1 antibody. **A.** The upper panel shows an area at low magnification (100x) with the tumour (right side) and the adjacent-to tumour (left side) tissues. The bottom panel shows detail at a higher magnification (200x): non-neoplastic ductal cells strongly express CHL1, while unstructured tumoral cells do not show CHL1 expression. **B.** High magnification (630x) pictures of a non-neoplastic, adjacent-to-tumour and tumoral tissue, showing the cytoplasmic pattern of CHL1 expression. All of the images were acquired using a Leica DMD 108 digital microscope (Leica, Wetzlar, Germany). **C.** CHL1 protein expression was explored by immunofluorescence in cultured immortalised but non-neoplastic mammary cells (HBL-100 cells). Green, red and blue stained CHL1, actin filaments and the nuclei, respectively. Images were captured at 400x magnification with a Leica TCS SP5 laser scanning microscope (Leica, Wetzlar, Germany).

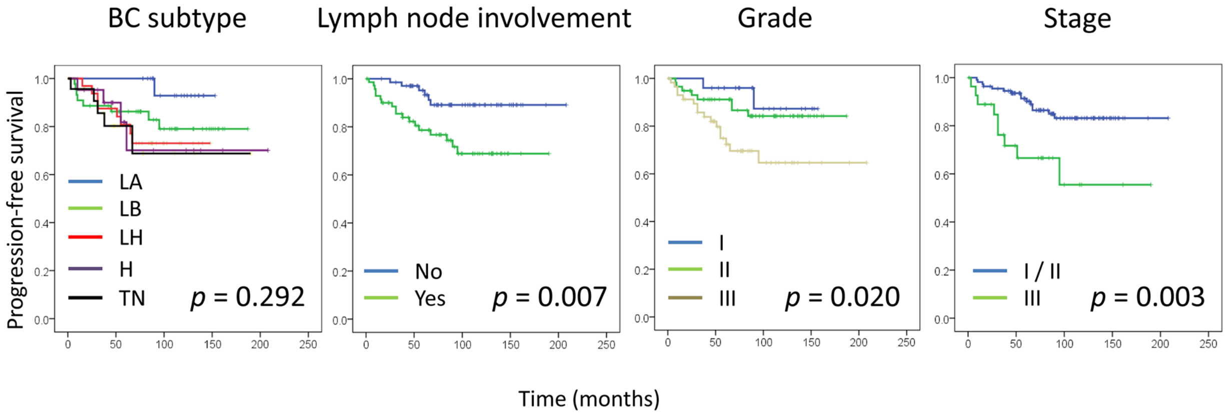

Supplementary Figure 4: Clinical value of important factors in BC prognosis. Associations between progression-free survival and BC subtype (LA: Luminal A-like; LB: Luminal B-like/HER2-negative; LH: Luminal B-like/HER2-positive; H: HER2-positive (non-luminal); TN: triple-negative), lymph node involvement, histological grade and stage were analysed in our series of 142 BC patients.

Supplementary Table 1: Pathological and clinical characteristics of our BC patient series

Feature	Frequency (%)
**BC subtype**	
LA	20/142 (14.1)
LB	44/142 (31.0)
LH	33/142 (23.2)
H	21/142 (14.8)
TN	24/142 (16.9)
**Grade**	
I	25/142 (17.6)
II	59/142 (41.5)
III	58/142 (40.8)
**Lymph node involvement**	
No	68/139 (48.9)
Yes	71/139 (51.1)
**Stage**	
I	49/138 (35.5)
IIA	34/138 (24.6)
IIB	27/138 (19.6)
IIIA	19/138 (13.8)
IIIC	9/138 (6.5)
**Age** (years)	Mean: 60<br>Range: 30-95
**Tumour size** (cm)	Mean: 2.2<br>Range: 0.3-10
**Progression-free survival** (months)	Mean: 82.9<br>Range: 1-208
No	115/141 (81.6)
Yes	26/141 (18.4)
**Overall survival** (months)	Mean: 86.9<br>Range: 1-208
Exitus	27/140 (19.3)
**Chemotherapy**	
No	49/138 (35.5)
Yes	89/138 (64.5)
**Hormone therapy**	
No	43/136 (31.6)
Yes	93/136 (68.4)

For the BC subtype: LA, Luminal A-like; LB, Luminal B-like/HER2-negative; LH, Luminal B-like/HER2-positive; H, HER2-positive (non-luminal); TN, triple-negative.

Supplementary Table 2: Primers used for pyrosequencing of the *CHL1* gene in 3 CpG sites

	CpG1	CpG2 and CpG3
**Forward primer**	TTTTTAAATGAAGGAAAGT AAGAAGATAAT	GTAATGGGAGAAAAGTAGATTGG
**Reverse primer**	[Btn]CCAATCTACTTTTCTC CCATTACT	[Btn]ACAAAAAACCAAACCAAAA ACTTTAATC
**Sequencing primer**	GTATATGGTATTATATTTT TTTAAG	ATTTGTGTGTGTAATATGAA

They were designed using PyroMark Assay Design software from Qiagen (Hilden, Germany).
